# The Book World of Medicine and Science

**Published:** 1902-09-20

**Authors:** 


### The Book World of Medicine and Science.

Protoplasm: Its Origin, Varieties and Functions. By John W. Hayward, M.D. (Bristol: John Wright and Co. 1902. 51 pages. 1s. 6d. net.)

This book illustrates the difference in value which exists between a paper when it is read before a scientific society and the same when printed in book form. Dr. Hayward's paper was probably most interesting to listen to as it is an agreeable resumé of well-known scientific facts and speculations concerning matter in general and protoplasm in particular. He arranges his materials so well that we pass in gradual stages from the consideration of the innate properties of pre-atomic matter to the functions of the central grey substance. It was worthy of being printed in pamphlet form as a souvenir of an interesting occasion. But, in the form of a book, it is difficult to divine the class of readers for whom it is intended. It would not be of particular interest to medical men because the physiology which it contains is elementary and familiar, and we should think that other scientists would find in it nothing with which they were not well acquainted; while it would be difficult for anyone who had not had some amount of scientific training, to understand the book. If the book were intended for the general public, an effort should have been made to accompany many of the statements with explanations, and such vague expressions as "cosmic activity" should have been avoided.

Clinical Lectures on Neurasthenia. By Thos. D. Savill, M.D., Physician to the West End Hospital for Diseases of the Nervous System, etc. Second Edition. (London: Henry J. Glaisher. New York: Wm. Wood and Co. 1902. Pp. 171. 5s.)

Dr. Savill's book is a sterling effort to treat neurasthenia in a scientific spirit, and to bring it into line with other diseases. This is most fortunate, for, as the author says, the disease is very common at the present time, and its victims suffer great mental distress and may become insane. It is to be hoped that there are not many medical men in England who consider the manifestations of neurasthenia to be "idle fancies" on the part of the patient, but if there be any, they will do well to read this book. The first lecture is particularly good. In it the causes of neurasthenia are divided into six groups, which are (1) structural changes, some perhaps undiscoverable with our present means of investigation; (2) toxins introduced into the body from without; (3) toxins manufactured within the body; (4) defective nutrition of the nerve tissues, as in anæmia; (5) deficiency or excess in the quantity of blood flowing through an organ; (6) increased reflex irritability. The rest of the book is, also, interesting, and the author emphasises the importance of a careful search on the part of the physician for some organic affection by quoting two cases of neurasthenia, in one of which an error of refraction was found, and in the other a movable kidney. Both of these affections were, fortunately, capable of being relieved, and in both the neurasthenic symptoms disappeared when the local trouble was treated. We agree with the author that many cases are caused by dyspepsia, and that a very large number of the phenomena are due to irregular action of the vasomotor nerves. He does not appear to have seen any cases in which the arterial tension was unusually high for long periods, but such cases are not at all uncommon. Probably, in these cases, there are times in the day or night, when the high tension passes off and the tension becomes low.

The Hair and its Diseases, including Ringworm, Greyness and Baldness. An Introductory Handbook by David Walsh, M.D.Edin. Medical Monograph Series No. 6. (London: Baillière, Tindall and Cox. 1902. Price 2s. 6d. net.)

Following out the plan adopted in this "Series," Dr. Walsh here gives us a good general outline of the diseases of the hair, an outline which well serves its purpose as an introduction to the subject. After a few remarks upon evolution, literature, barbers, hairy races, and other cognate subjects, we come to a chapter on the anatomy and physiology of the hair, followed by one upon its hygiene. Dr. Walsh is evidently not very fond of the hairbrush, regarding it as a common source of infection, not merely in the nursery, but at clubs, hotels, or the houses of friends, and he very properly points out how easy it would be for most people to keep themselves tidy as to their hair by the use of a small metal pocket-comb. All combs should be made of metal so as to bear sterilisation. "If a child's hair be kept reasonably short there will be little or no need for the use of a brush. The nursery comb should be plunged daily into boiling water, and one should be provided for each child." A point of considerable importance on which Dr. Walsh insists is that it is not merely infection from others that one has to guard against, but re-infection from oneself. "If the brush and comb of a seborrhœic patient be neglected they become charged with micro-organisms and reinfect the scalp. Under these circumstances such a patient may be keeping up the unhealthy process in his scalp for two-score years, until nature retires from the contest and leaves a bald scalp which is no longer capable of infection." A very considerable portion of the book is devoted to a consideration of "shedding of the hair and baldness," the local causes of which are divided into three classes:—(1) Mycoses, the gross fungi; (2) bacterial invasions; and (3) animal parasites. The bacterial invasions include alopecia areata, seborrhœa, and eczema; and following the latter heading, but apparently unclassified, come psoriasis, sycosis, lupoid sycosis, and tinea tarsi. The preventive treatment of seborrhœa consists, we are told, in the systematic washing of the scalp, combined with disinfection of the brush and comb; while in the remedial treatment reliance seems to be placed upon the use of various stimulant and antiseptic preparations, in addition, of course, to repeated washing. Schmolle's soap, or an alcoholic solution of green soap, as advised by Hebra, are the kinds of soap recommended for the purpose. Dr. Walsh's favourite prescription for a stimulating antiseptic lotion is the following:—℞ Resorcini, ʒi; glycerini acidi borici, ʒii; spiritus rectificati ad ℥viii. Misce: fiat lotio. The book will be found a useful one, practical and to the point, although it does not, as indeed it does not profess to, go very deeply into the subject.

